# An exploration of perceptions of flourishing and social determinants of health among medical, physician assistant, and nurse practitioner students: A mixed methods study

**DOI:** 10.1371/journal.pone.0343630

**Published:** 2026-02-25

**Authors:** Stephanie Neary, Benjamin Doolittle, Martina Mueller, Michelle Nichols

**Affiliations:** 1 Department of Internal Medicine, Section of General Internal Medicine, Physician Assistant Online Program, Yale University, New Haven, Connecticut, United States of America; 2 Department of Internal Medicine, Section of General Internal Medicine, Yale University, New Haven, Connecticut, United States of America; 3 College of Nursing, Medical University of South Carolina, Charleston, South Carolina, United States of America; 4 Department of Public Health Sciences, Medical University of South Carolina, Charleston, South Carolina, United States of America; 5 Department of Health Sciences and Research, College of Health Professions, Medical University of South Carolina, Charleston, South Carolina, United States of America; Bilawal Medical College, Liaquat University of Medical and Health Sciences, PAKISTAN

## Abstract

The purpose of this study is to describe individual perceptions of flourishing among medical (MD), physician assistant (PA), and nurse practitioner (NP) students and the relationship between these perceptions and social determinants of health (SDOH). MD, PA, and NP students from two universities were recruited to participate in this explanatory sequential mixed-methods study. The quantitative phase took place between August and October 2023, and findings were used to purposively recruit participants for interviews that were conducted in January and February 2024. The survey included the WellRx and the Secure Flourish Index (SFI); participants applied a percentage weight (0–100%) to each of the six SFI domains of flourishing based on their perceived relative importance to their individual flourishing. Survey participants were stratified into three SDOH risk levels based on WellRx score; descriptive statistics and ANOVA testing were performed (alpha = 0.05). Interviews explored definitions of flourishing, perceptions of flourishing domains, and how SDOH needs relate to flourishing. A total of 280 of 1820 eligible students (15.4%) completed the survey, and 34 students across all three established SDOH risk groups were interviewed. Results demonstrated a significant relationship between SDOH factors and flourishing perceptions. Low-risk group students had an average SFI score that was over 15.0 points greater than those in the high-risk group (M88.0(SD ± 14.4) versus M72.7(SD ± 13.5); p < .001). Perceived relative domain importance to flourishing significantly varied by SDOH risk level for multiple domains. Participant definitions of flourishing included elements of personal growth and a balance of happiness and success; interviews revealed that students in all SDOH risk levels are experiencing financial strain that negatively impacts flourishing across SFI domains. Perceptions of flourishing and the relative importance of established flourishing domains are highly influenced by social determinants of health and living experiences. Educators should consider individual perspectives and values when measuring flourishing and planning wellness interventions.

## Introduction

Poor mental health is a leading cause of attrition among medical (MD), physician assistant (PA), and nurse practitioner (NP) students [1–3]. Depression risk and suicidal ideation have been found to increase rapidly in the months following matriculation [4]. In addition to increased rates of attrition, provider burnout and poor mental health have been found to increase medical errors and adverse patient outcomes [5].In response, accreditation bodies have modified policies in recent years to require training on multiple wellness related topics including provider burnout and resilience [6–8]. While these efforts represent important steps towards student wellness, a critical gap exists in understanding how students perceive flourishing and how their living experiences shape these perceptions. Without this understanding, wellness interventions may not fully align with students’ needs and values. There remains a paucity of data on a strengths-based approach to this mental health crisis, with many existing efforts focusing on burnout mitigation rather than the promotion of flourishing [9–11].

Flourishing is an interdisciplinary concept that spans multiple fields including psychology, sociology, and theology [12]. Flourishing has been recently operationalized by the Secure Flourish Index (SFI), which proposed six different domains of flourishing including Happiness and Life Satisfaction, Mental and Physical Health, Meaning and Purpose, Character and Virtue, Close Social Relationships, and Financial and Material Stability [13]. While living in accordance with individual meaning and purpose has been established as a foundational element of flourishing, a recent study found that students have varied perceptions on the relative contribution of meaning and purpose, as well as the remaining SFI domains, to their overall ability to flourish [12–15]. This raises an important question of how individual and environmental factors may shape the definition of flourishing as well as perceptions of individual flourishing among students. Furthering our understanding of these individual values and perceptions, paired with living experiences, may strengthen the development of wellness interventions and creation of learning environments that promote flourishing.

Flourishing among students is also largely related to social and economic needs, with lower flourishing in students with more unmet needs; students with more unmet needs were also more likely to have considered leaving training [13,16,17]. While individual factors such as grit, avoidant coping, and affect have all been linked to student flourishing, less is known about the role these living experiences play in constructing a personal definition of flourishing or how they shape the relative value of the flourishing domains [15,18]. Additionally, medical and nursing training remains focused on competency-based education [8,19-20], promoting learners with high academic achievement with limited consideration of individual well-being, despite known relationships between well-being and attrition, job satisfaction, and patient outcomes [1-2,5,21]. While wellness interventions and support are typically available to learners, wellness is generally not a factor in program progression and there remains a significant gap in access to necessary mental and physical health care among these students [16,22]. Further understanding of these basic needs among students, and how they relate to and shape individual flourishing, allows for the development of expanded support systems beyond the classroom and the promotion of equity through targeted interventions.

The purpose of this study was to describe individual perceptions of flourishing among MD, PA, and NP students and the relationship between these perceptions and social determinants of health (SDOH).

## Methods

### Study design and quality

We used an explanatory sequential mixed-methods approach to further explore the factors associated with the perceived relative importance of flourishing domains as established by the Secure Flourish Index (SFI) [23]. This included a quantitative cross-sectional survey followed by qualitative semi-structured interviews. This approach first enabled quantitative identification of relationships between SDOH and domains of flourishing and results were used to inform the interview participant selection, interview protocol, and *a priori* codebook. The following interviews allowed for qualitative exploration into the underlying perceptions and living experiences shaping these quantitative findings. Study quality and rigor was maintained using validated instruments, purposive interview sampling, and cross-coding of qualitative data. Additionally, consensus discussions were employed, and data triangulation was achieved through integration of quantitative and qualitative findings. The results are presented in an integrated design using the SFI domain scores, social determinants of health risk level, and representative quotes, allowing for expanded interpretation of the findings [12,24]. This study is part of a larger project, and detailed quantitative methods have been described in prior publications [14–16]. The study was reviewed and considered exempt by the Institutional Review Boards at the Medical University of South Carolina (#00129125) and Yale University (#2000035757).

### Study participants and setting

Students enrolled in MD, PA, and NP programs at two universities were initially recruited to participate in a cross-sectional quantitative survey, including a combination of online and campus-based students at both institutions. Both Master of Science in Nursing and Doctor of Nursing Practice students were included. While MD-PhD students were included, PhD in Nursing Science students were excluded as there is no clinical component to their program.

### Procedure for quantitative survey

Survey recruitment used a combination of emails, flyers, and announcements during live in-person and online sessions between August 6 and October 9, 2023. The target survey response was 310, using a 95% confidence interval with a 5% margin of error (estimated population size of 1820 students) [25,26]. The survey was delivered through REDCap, a secure survey platform [27]. Participants agreed to a statement of informed consent prior to completing the survey which included demographics, considerations for leaving training, and multiple validated instruments including the SFI (flourishing) and WellRx (SDOH) [12,27]. A novel, self-weighted approach to the SFI was also used to capture individual perceptions of relative importance of flourishing domains. Students were asked to divide 100% among the six SFI flourishing domains based on how much they perceive the domain contributes to their overall ability to flourish, with a higher percentage indicating a greater contribution. A complete description and analysis of this approach as compared to the traditional SFI scoring approach has been previously described [14–15]. Students were provided a five-dollar e-gift card for their participation in the survey. The complete survey can be found in S1 File.

### Instrument scoring

SDOH needs were measured using the WellRx, which was modified to add one question inquiring about health care access and to remove one question on education access [27–28]. The WellRx contains 11 yes/no questions; a greater sum suggests a greater number of unmet social and/or economic needs. The SFI measured flourishing using 12 11-point Likert scale questions, with two questions in each of six flourishing domains [12]. Greater scores indicate greater domain or overall flourishing. For cases missing one SFI response, the paired domain question score was imputed for the missing response. For cases missing one WellRx response, a score of zero was imputed for the missing question. Cases missing more than one SFI or WellRx response, or where domain percentage weights did not sum to 100%, were excluded.

### Statistical analysis

Descriptive statistics were used to analyze demographic characteristics. Students were assigned to one of three SDOH risk levels based on WellRx total score (low risk: 0; moderate risk: 1–2; high risk: 3+). Means and standard deviations were calculated for the total SFI and the SFI domain weights and one-way analysis of variance (ANOVA) was performed to compare these means across the three SDOH risk level, using the nonparametric Kruskal-Wallis test when necessary. SPSS version 28 was used for data analysis with significance set to an alpha of 0.05.

### Procedure for qualitative interviews

We stratified students indicating interview interest on the survey into the three established SDOH risk groups, ordering them from highest to lowest SFI score within each group. We used purposive sampling within each group to invite students via email to participate in interviews beginning simultaneously at the top and bottom of each SFI score range, working towards the center from both directions across all groups at once. We set a target of 36 interviews (12 per SDOH risk strata), with the final number determined by data saturation. Interviews were scheduled for one hour and conducted using a secure online video conferencing platform [Microsoft Teams] to allow participants to join from the location of their choice. One researcher [SN] and the participant were the only individuals in the interview; while video and audio were used for the interviews, only audio was captured. The researcher kept field notes following each interview. Interviews occurred between January 12 and February 21, 2024, and no repeat interviews were conducted. Students were provided a $25 e-gift card for their participation in the interview.

The interviewer [SN] is a PhD candidate at one institution included in the study, where she has no interaction with students in the included programs. She serves as faculty at the second included institution; she is not responsible for course grades for any study participants. Her university affiliations and roles were disclosed in the consent statement prior to the start of each interview.

### Interview guide

Interviews followed a semi-structured format and *a priori* prompts were developed with consideration of the background literature and the quantitative findings by all four researchers. Prompts covered topics of perceptions of flourishing, social and economic needs, grit, and coping style. All interviews began with reading an informed consent statement and verbal consent to participate; open-ended questions were asked with follow up questions for clarification when needed. The Social Ecologic Model (SEM) was used as the guiding framework for interview question development allowing for the exploration of multi-level factors and creating a holistic view of student flourishing at each level [individual, interpersonal, organizational, community, public policy] [29]. At the conclusion of the interview, each participant was asked to share any additional thoughts they had that were not otherwise captured. The complete interview protocol can be found in **S2 File**.

### Interview analysis

Interview recordings were anonymized then transcribed verbatim using automated transcription [Ubiqus]; transcripts were read and corrected by SN using the recorded interview for verification and were not returned to the participant. De-identified transcripts were uploaded to MAXQDA for analysis. Using an *a priori* codebook, a thematic descriptive approach was used to analyze the transcripts, adding to and modifying the codebook as needed as the analysis evolved to capture new themes and subthemes. Initial coding was conducted by one researcher [SN], and four (11.7%) interviews were duplicatively coded by a second researcher [MN]. Discrepancies were discussed in multiple face-to-face meetings, and a consensus on final codes and operant definitions were established. All transcripts were then re-read and coded considering the final code adjustments by one researcher [SN]. Codes were collated and themes were reviewed to verify relationships with the data for each code; an audit trail was maintained. Participant quotes supporting each theme and subtheme were extracted from the transcripts and data saturation was achieved. Results were framed in part by the social determinants of health, providing a novel perspective on existing definitions of flourishing. Participants were not asked to provide feedback on the findings. The themes and subthemes used to organize the results are displayed in Box 1.

**Box 1. Outline of themes and subthemes**

**Theme 1: Defining Flourishing**

*Multi-faceted thriving*
*Personal growth*
*Balancing happiness and success*

**Theme 2: Relative Domain Contribution to Flourishing**

*Domain interaction*
*Domain security*
*Current domain priority and impact*
*Consideration for others*

**Theme 3: Social Determinants of Health**

*Health Care Access and Quality*
*Economic Stability*
*Neighborhood and Built Environment*
*Social and Community Context*
*Education Access and Quality*

## Results

Of the 1820 eligible students, 393 began the survey (21.6%). A total of 280 out of 1820 students (15.4%) completed the WellRx, SFI, and SFI domain weighting questions and are included in the quantitative analysis. Qualitative data saturation occurred after 29 interviews, and an additional five already scheduled interviews were then conducted, totaling 34 interviews. No participants withdrew from the study. Interview participants included eight men (23.5%) and 26 women (76.5%). Sixteen were PA students (47.1%), nine were MD students (26.5%), and nine were NP students (26.5%). As students were purposively sampled based on SDOH risk level and SFI scores, interviewee demographics are expected to differ from the survey cohort. However, we believe that purposively sampling for these factors provided a stronger ability to capture a more diverse student perspective than attempting to match on participant demographics, which were previously shown to have minimal impact on overall flourishing [15]. Complete demographics for survey and interview participants are reported in Table 1. Throughout the results, participant quotes are accompanied by the interview number, SDOH risk level group (low, moderate, high), and total SFI score.

**Table 1:** Demographic and other personal factors data for survey (N = 280) and interview (N = 34) participants.

	Survey Participants^a^		Interview Participants^a^			
	n	(%)	n		(%)	
**Training Profession**	**280**		**34**			
*Medical Doctor*	95	33.9	9		26.5	
*Nurse Practitioner*	59	21.1	9		26.5	
*Physician Assistant/Associate*	126	45.0	16		47.1	
**Gender Identity**	**275**		**34**			
*Man*	61	22.2	8		23.5	
*Woman*	211	76.7	26		76.5	
*Other/Prefer not to disclose*	3	1.1	0		0.0	
**Race** ^b^	**280**		**34**			
*White*	197	70.4	22		64.7	
*Black or African American*	17	6.1	2		5.9	
*American Indian or Alaskan Native*	5	1.8	0		0.0	
*Asian*	49	17.5	8		23.5	
*Native Hawaiian or Pacific Islander*	0	0.0	0		0.0	
*Prefer not to answer*	17	6.1	2		0.0	
**Hispanic, Latinx, or Spanish**	**274**		**34**			
*Yes*	16	5.8	0		0.0	
**Year in training**^c^	**275**		**34**			
*< 3 months*	59	21.5	5		14.7	
*3 months – 1 year*	67	24.4	8		23.5	
*Year 2*	91	33.1	10		29.4	
*Year 3*	32	11.6	7		20.6	
*Year 4*	26	9.5	4		11.8	
***Relationship status***	**275**		**34**			
*Single*	172	62.5	22		64.7	
*Married, Domestic Partner, or Civil Union*	96	34.9	12		35.4	
*Separated, Divorced, or Widowed*	7	2.6	0		0.0	
***Parental Undergraduate Degree Status***	**274**		**34**			
*Yes, both parents*	158	57.7	20		58.8	
*Yes, one parent*	52	19.0	6		17.6	
*No, neither parent*	64	23.4	8		23.5	
***Seriously considered dropping out in the past 6 months?***	**275**		**34**			
*Yes*	41	14.9	4		11.8	
***Factors influencing drop out consideration*** ^*d*^	**41**		**4**			
*Personal mental health*	27	65.9	3		91.2	
*Financial stress*	23	56.1	2		50.0	
*Family stress*	16	39.0	1		25.0	
*Lack of program connection*	11	26.8	1		25.0	
*Difficulty of course work*	10	24.4	1		2.9	
*Personal physical illness*	4	9.8	0		0.0	
*Something else*	6	14.6	0		0.0	
	**n**	***M(SD)***	***Median*** ***(Q1:Q3)***	**n**	***M(SD)***	***Median*** ***(Q1:Q3)***
**Age, years**	**269**	28.7(6.5)	26.0 (24.0:32.0)	**34**	29.9(5.3)	29.5 (25.8:34.0)
**Number of legal dependents**	**267**	0.5(1.0)	0.0 (0.0:0.0)	**33**	.4(1.1)	0.0 (0.0:0.0)
**Secure Flourish Index** ^e^	**280**	84.0(15.0)	86.0 (73.3:95.0)	**34**	80.9(20.7)	79.0 (62.5:101.5)
**WellRx** ^e,f^	**280**	1.1(1.3)	1.0 (0.0:2.0)	**34**	1.5(1.6)	1.0 (0.0:3.0)

^a^Interview participants are included in the survey response data as these students also completed the survey.

^b^Race and factors contributing to drop out consideration were asked in a ‘select all that apply’ format so total percentage equates to more than 100%.

^c^Year in training was reflects year at the time of the survey, which was approximately 6 months prior to the interviews.

^d^Participants were asked to select all factors contributing to their drop out consideration.

^e^Instrument name (possible score range): Secure Flourish Index (0–120); WellRx (0–11).

^f^One question was added to the WellRx asking about access to physical and mental health care to meet the Social Determinants of Health domain of health care access and quality. One question on access to education was omitted as all students are currently enrolled in a graduate or doctoral program.

### Theme 1: defining flourishing

The varying perspectives on defining flourishing was a major theme for participants across all three SDOH risk levels. Within these provided definitions, multiple subthemes were identified, with a sense of thriving across multiple facets of life, opportunities for and experiences of personal growth, and achieving a balance between happiness and success being consistently present in the provided definitions.

#### Multi-faceted thriving.

Participants in all risk groups expressed the need for not only survival but thriving in multiple areas of their life to flourish. For many, this definition captured their roles as a student but also other pieces of their identities and personal lives, such as their relationships and their physical and mental health. However, no students noted the need for financial thriving in this definition:

*Each part of me would be operating at its best. (#15, Low, SFI:80)*

*Doing well in multiple facets of life, I would say, and not even just doing well, I would say exceeding, you know, doing well personally, interpersonally, educationally. (#33, High, SFI:61)*

This multi-faceted perspective to defining flourishing also left many participants seeking an equally broad sense of balance in these areas. While thriving may be present in one aspect of life, such as academics, to flourish, they shared that this academic flourishing must not be at the expense of thriving in other facets of life:

*I think flourishing for a student in a healthcare professional training program would be able to learn as much in the program as possible, while also being healthy and happy, so learning in the program, but then also not sacrificing kind of those personal components as well. (#20, High, SFI:76)*

#### Personal growth.

While individual definitions of flourishing varied between participants, reference to personal growth was central to this definition for over one-third (13/34; 38.2%) of students interviewed. This included both personal and professional growth, as well as learning from current experiences:

*I think that a term like flourishing implies that there is both happiness and forward growth. So, you – it’s not just about being stable, it’s about transforming your happiness into something more, and continuing to do that. Not just staying static. (#16, Low, SFI:103)*

*But I think flourishing has a beginning, but it doesn’t really have an end. (#12, High, SFI:53)*

Forward motion may come from an intrinsic drive for continued growth, but students also noted the role of support in this journey of growth and flourishing:

*Being nurtured in the environment, and to grow in a way that maximizes potential. (#12, High, SFI:53)*

#### Balancing happiness and success.

Happiness was most often described as fleeting and under intrinsic control, such as enjoying an activity in the moment. Success was more often described in terms of achievement of external benchmarks, such as graduating, getting a desired job, or receiving an award. However, over half of participants (19/34; 55.9%) stated or agreed that flourishing is a balance of happiness and success:

*I think in my head, flourishing is both happiness and success, like it’s contingent on there being both. (#32, Low, SFI:97)*

*I think they kind of go hand in hand. I don’t think you can flourish without having some amount of happiness and success in your life. (#2, Moderate, SFI:46)*

**Fig 1:**
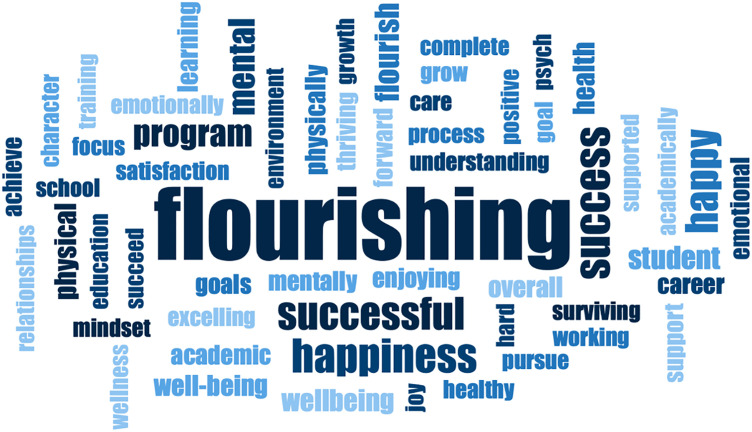
Word cloud of *Defining Flourishing* theme This word cloud presents the most commonly words used to define flourishing by interview participants. More frequent word use is indicated by larger word font, while less frequently used words appear in small font.

Overall, the survey participants have a mean SFI score consistent with flourishing (84.0(SD ± 15.0) of a possible 120 points; Table 1). However, exploration of the strategies and considerations for applying domain weights allows for a deeper understanding of how the SFI can be adapted to fit these individual definitions and produce a flourishing score that is more consistent with and reflective of these individual perceptions. Fig 1 presents a word cloud representing the *defining flourishing* theme based on frequency of word use.

### Theme 2: Domain weighting

While reflecting on the percentage weights applied to each SFI domain during the survey, interview participants considered how flourishing in one domain may impact the ability to flourish in other domains, how secure they feel in a domain, the influence of recent life events, or how their flourishing impacts those around them.

#### Domain interaction.

Participants described relationships between domains that either promoted or detracted from their ability to flourish; however, which domains were perceived to overlap or interact varied greatly between participants:

*Having health will help engender those other items, and if I don’t have health, it would be hard to have those other pieces also in place. (#15, Low, SFI:80)*

*I remember looking at that and like feeling confused about the happiness category because I was like, I feel like all of these are part of my happiness and like I wouldn’t see happiness as something separate. (#20, High, SFI:76)*

#### Domain security.

The subtheme of domain security emerged throughout the interviews, as participants identified flourishing domains where they currently feel fulfilled or their needs were being met. However, there were differing perspectives on how these more secure domains were weighted. Some participants viewed these domains as being secure in the background, and therefore did not impact their ability to flourish, so they weighted them with a lower percentage:

*And thinking back on that list in terms of delayed gratification, purpose, finances, and all of that, I guess I’m realizing the reason why I ranked them kind of lower is those are things that those are things that I feel like I already have, if that makes sense. Those are things that I actually feel very comfortable and secure about. (#22, Moderate, SFI:71)*

Conversely, some students felt the secure domains created a foundation for flourishing, and therefore weighted them with a greater percentage:

*I’m very happy. I think I also am generally a happy person. So it’s hard for me not to be happy, which is another reason why it ranks pretty high in my flourishing. (#32, Low, SFI:97)*

This difference in the approach to weighting the same foundational idea of domain security emphasizes the need to consider relative domain importance while evaluating flourishing.

#### Current domain priority and impact.

Variations in relative domain contribution to overall flourishing were also found to be influenced by recent life events, reinforcing the idea that flourishing is a dynamic life course rather than a fixed state of being. Recent life experiences were shared as contributors to the current definition of flourishing as well as the relative domain weights applied in the survey:

*I think the main reason I put that one [Mental and Physical Health] up there is, uh, around the time that we were filling out this survey, um, I had a number of family members that were having health scares, um, or were making end of life treatment decisions for, you know, grandparents and things like that. (#34, High, SFI:74)*

However, others weighted domains based on how much impact they thought improvements in the domain would make in their overall ability to flourish, weighting domains with a bigger perceived impact with a higher relative percentage to domains with a smaller perceived impact:

*Or, like, which ones did I think would make more of a difference if they were added in to increase my bang for their buck, I guess. (#1, Low, SFI:47)*

#### Consideration for others.

Defining flourishing was found to be highly personal but through the interviews, many participants shared how they considered the impact their flourishing has on those around them when applying domain weights:

*I think I chose those two, like, the individual character as well as, like, social relations, because it highlights two things. One of the two principles that I’ve carried with me personally is the balance between individualism as well as, like, community or collectivism. (#24, Low, SFI:100)*

*I definitely think I’m heavy on what my purpose is and like what am I contributing to my community and my people. So I think about it daily, like how is what I’m doing contributing or helping others. (#29, Moderate, SFI:64)*

Table 2 presents four separate interviews with varying SDOH risk groups and SFI scores. Additionally, representative quotes are displayed to further contextualize the domain weighting process. These cases show both concordance [#29, Character and Virtue], where SFI domain score and perceived weight align, and discordance, where SFI domain score and perceived weight do not align [#9, Financial and Material Stability].

**Table 2:** Case examples of domain weighting strategy and supporting quotes.

Interview #29, SFI Score ^a^: 64, WellRx Score: 1 (Moderate SDOH Risk Level)^b^			
SFI Domain	SFI Domain Score (0-10)	Weighted %^c^ (0-100%)	Exemplary Quotes
Happiness and Life Satisfaction	3.5	20%	*“…As long as if I fulfill my purpose and I portray myself as being a great person, then that happiness and life satisfaction will come.”*
Mental and Physical Health	5	10%	*“With school, like I, the way my day runs, I don't have time to like really go and exercise… So, I just feel like I don't prioritize my physical health like I would versus not being a student.”*
Meaning and Purpose	7	30%	*“I definitely think I'm heavy on what my purpose is and like what am I contributing to my community and my people. So, I think about it daily, like how is what I'm doing contributing or helping others.”*
Character and Virtue	7.5	30%	*“So, I am always worried about how I present myself and what my character is and how it's presented to other people. And then just being like virtuous in a sense of like, am I true to who I am? Am I a person of my word and that kind of thing?”*
Close Social Relationships	2.5	5%	*“I do feel a lot better when I do have a lot of social interaction, but I'm also like an introvert as well.”*
Financial and Material Stability	6.5	5%	*“I don't feel like the money that I have contributes to me flourishing in life. I think it's other factors.”*
**Interview #22, SFI Score** ^a^**: 71, WellRx Score: 1 (Moderate SDOH Risk Level)**^b^			
**SFI Domain**	**SFI Domain Score** **(0-10)**	**Weighted %** ^c^ **(0-100%)**	**Exemplary Quotes**
Happiness and Life Satisfaction	6.5	50%	*“I think being happy in the work that you do will bring a lot more flourishing in general in the long term than success over a status or one event or something you could do.”*
Mental and Physical Health	5.0	10%	*“There's a good portion of my friends who have failed a few exams because there are a lot of stuff going on in their life, whether that be health, chronic illnesses, or things like that. And I guess because I don't have any of those issues, they're ranked inherently a lot lower.”*
Meaning and Purpose	6.5	10%	*“I think it's very much okay for medical students at this stage to still be looking for their meaning and purpose.”*
Character and Virtue	7.0	10%	*“In terms of delayed gratification, purpose, finances, and all of that, I guess I'm realizing the reason why I ranked them kind of lower is those are things that those are things that I feel like I already have”*
Close Social Relationships	7.0	15%	*“I have a few really close friends that I talk to them genuinely all the time, starting from the moment that I wake up to when I go to sleep. I have a lot of people that I'm happy to have conversations with, that I would say they're pretty okay friends….And then, I feel like I'm relatively okay acquaintances with a lot of my school peers as well…So, I would say socially I'm pretty happy with the friends that I have here.”*
Financial and Material Stability	3.5	5%	*“I don't come from a super wealthy family and relative to my peers I would say my family is probably at the lower end. And I think that has – that inherently gives me a lot of appreciation for the things that I have now.”*
**Interview #23, SFI Score** ^a^**: 95, WellRx Score: 2 (Moderate SDOH Risk Level)**^b^			
**SFI Domain**	**SFI Domain Score** **(0-10)**	**Weighted %** ^c^ **(0-100%)**	**Exemplary Quotes**
Happiness and Life Satisfaction	7.0	20%	*“I'm a really like goal-oriented person I would like to think if I wasn't a student, I would have a different goal like or like I would have like a different goal but it would still be oriented towards happiness.”*
Mental and Physical Health	8.0	20%	*“Mental and physical health…I think that goes along with just being like a health care provider …if you're not feeling well and being in your best physical state you know it's really hard to do what you need to do.”*
Meaning and Purpose	7.5	20%	*“Over the last couple of years like I've just really learned that about myself like it's really important to have like goals and like a purpose behind life.”*
Character and Virtue	9.5	15%	*“A lot of times the experiences that are helping you flourish, they may not be like the most positive. They might be hard. They might be like tough experiences, but in the long term, they are going to help you succeed and like hopefully make you happy in the future.”*
Close Social Relationships	7.0	10%	*“I have like fewer friends that are close um rather than like a lot of friends um and I think I've just been like hurt a lot in the past and so I just don't necessarily, I don't know, I'm very independent and I just don't necessarily rely on a lot of people.”*
Financial and Material Stability	8.5	5%	*“My family's…first generation immigrants and they've always kind of struggled monetarily…so um like I'm always kind of prepared to do that.”*
**Interview #9, SFI Score** ^a^**: 63, WellRx Score: 3 (High SDOH Risk Level)**^b^			
**SFI Domain**	**SFI Domain Score** **(0-10)**	**Weighted %** ^c^ **(0-100%)**	**Exemplary Quotes**
Happiness and Life Satisfaction	1.0	10%	*“I think that I'm just so preoccupied with like trying to like do what is best long-term…Instead of just, “Oh, well that makes me happy, I'm going to do it,” because sometimes that is to be misguided, selfish, and like I said, it's fleeting happiness and then that leaves you feeling empty.”*
Mental and Physical Health	5.5	20%	*“But just the nature of just how accelerated it is there's no time to be burned out. So, you kind of just sped up like, like you keep walking and while you're wiping off your tears. Like it's, you know, we don't have time for that.”*
Meaning and Purpose	5.0	10%	*“I think it's important ideally, but I don’t know and have time and the ability and the energy and the circumstance to be able to find our true meaning and purpose in this life. But I don't think it's a necessary requirement.”*
Character and Virtue	10.0	30%	*“You are the sum of the people that you keep around and everybody is good in their own way, but people who really prioritize like I'm going to be altruistic…character and virtue…those are pillars of my being. I think that that's definitely something that I have always, always prioritized.”*
Close Social Relationships	10.0	10%	*“It does speak to how I am socially fulfilled and maybe that was probably the reason why I ranked it so low is because it's not something that weighs on my mind. I am fulfilled there.”*
Financial and Material Stability	0.0	20%	*“Healthcare is not ethical…I’m going to be making six figures off people that like are going to have medical debt for a chronic illness they didn't ask for…I am for things like universal healthcare, I'm for those things. Money rules our lives in ways that it fundamentally should not and that leaches into our wellness.”*

Abbreviations: SFI: Secure Flourish Index; SDOH: Social Determinants of Health

^a^SFI total score has a possible range of 0–120 points.

^b^WellRx has a possible range of 0–11 points. SDOH risk groups were established using WellRx scores [low risk = score of 0; moderate risk = score of 1–2; high risk = scores of 3+].

^c^Participants were asked to apply a percentage weight (0–100%) to each domain that indicates how much they perceive the domain to contribute to their overall ability to flourish.

### Theme 3: Social determinants of health

Mean overall flourishing scores, domain specific flourishing scores, and percentage weight applied to each domain were calculated for survey participants in each of the three predetermined SDOH risk level groups (Table 3). Students in the low-risk group had an average total SFI score that was over 15.0 points greater than those in the high-risk group (88.0(SD ± 14.4) versus 72.7(SD ± 13.5); p < .001). Similarly, there was discordance between low- and high-risk groups in the Happiness and Life Satisfaction domain as low-risk students had both a higher domain flourishing score (p = .01) and applied a larger percentage to this domain (p < .04):

**Table 3:** Comparison of means of social determinants of health (SDOH) risk level based on WellRx score with Secure Flourish Index (SFI) scores and novel SFI domain weights (N = 280).

	SDOH Level^a^	n	M(SD)				p-value
**SFI Total Score**	Low	110	88.0(14.4)				<.001
	Moderate	137	83.5(14.5)				
	High	33	72.7(13.5)				
			**SFI Domain** **Scores** (**0-10)**		**SFI Domain Weights** (**0-100%)**		
		**n**	**M** **(SD)**	**p-value**	**M (SD)**	**p-value**	**Exemplary Quotes**
Happiness and Life Satisfaction	Low	110	7.2 (1.6)	.01	19.2 (7.3)	.04^+^	*Int. 5 (Moderate, SFI: 112) “So, it’s like a catch-22. Like, what do you do? You go to these events and blow off steam, or do you work?”* *Int. 9 (High, SFI: 63) “You know, “Oh well money can't buy happiness,” that's something rich people say to poor people.”*
	Moderate	137	6.8 (1.6)		21.5 (10.2)		
	High	33	6.3 (1.8)		16.7 (7.6)		
Mental and Physical Health	Low	110	6.9 (1.7)	<.01	20.7 (8.5)	.77	*Int. 29 (Moderate, SFI: 64) “I feel slightly guilty. I'm like, you know, I'm spending an hour at the gym, and I could have been spending an hour studying or something like that.”* *Int. 7 (High, SFI: 83) “It gets hard, I think, when your physical and mental health start to decline because this is now part of your life. It's easy to think, “If I get rid of it [school], then I will feel so much better.”*
	Moderate	137	6.4 (1.7)		20.0 (8.4)		
	High	33	5.8 (1.7)		19.8 (8.3)		
Meaning and Purpose	Low	110	7.8 (1.7)	.09^+^	16.4 (9.4)	.54	*Int. 6 (Moderate, SFI: 61) “I felt like I lost sight of the purpose a little bit during didactic year, but I feel like now that we're back kind of in the real-world seeing patients, interacting with providers, it definitely feels better now getting to see that side and not just being sitting in a classroom for, you know, six hours, eight hours a day.”* *Int. 29 (Moderate, SFI: 64) “I don't feel like I'm really carrying out my purpose right now… I don't have the free time like before being a student, I was able to volunteer like anywhere. I could help out at my church. Like I felt like I was really like contributing… in the back of my head, I'm like, okay, this is all leading up to me going towards my purpose.”*
	Moderate	137	7.6 (1.7)		15.9 (7.1)		
	High	33	7.1 (1.7)		14.6 (9.3)		
Character and Virtue	Low	110	7.6 (1.3)	.29	12.7 (5.9)	.82^+^	*Int. 30 (Low, SFI: 101) “If I like lose my focus on virtues and, um, building myself as a person, I've lost everything else.”* *Int. 12 (High, SFI: 53) “A person who has that character, built virtue in the best form, are other people, when you encounter them, you feel like you're walking in a garden. I love that, and I feel like that's so important for me. Like, when I meet people, that I will bring positive energy, make them feel like they are growing, make them feel like they are finding joy and positive, in that sense, and I feel like I'm still getting there. I'm not there yet.”*
	Moderate	137	7.7 (1.3)		13.1 (6.2)		
	High	33	7.3 (1.4)		15.0 (9.3)		
Close Social Relationships	Low	110	7.3 (2.0)	<.01^+^	16.8 (7.9)	.34	*Int. 30 (Low, SFI: 101) “A lot of my classmates, they come from all over the world really. And now this is kind of like the main community they have. They don't, they don't go back to their families very often. And so, for there to be community on campus is a huge deal.”* *Int. 6 (Moderate, SFI: 61) “I feel like PA school really like made me value just like where you are so stressed and you have so limited time, so like I don't care to have any of these superficial relationships or anything that feels that way.”*
	Moderate	137	7.0 (2.1)		15.6 (7.5)		
	High	33	6.0 (2.1)		15.0 (5.8)		
Financial and Material Stability	Low	110	7.1 (2.6)	<.001^+^	14.3 (8.2)	.01^+^	*Int. 32 (Low, SFI: 97) “Instead of I don't know… like spending an hour like doing a side job just to get extra money, I can spend that hour, like going to the gym, or like doing some personal well-being activity.”* *Int. 12 (High, SFI: 53) “I basically sacrificed my social relationship to get our financial childcare stability. That's the reason why we moved, to be closer to my in-laws.”*
	Moderate	137	6.3 (2.6)		14.0 (8.3)		
	High	33	3.9 (2.3)		19.0 (10.2)		

Abbreviations: SFI: Secure Flourish Index; SDOH: Social Determinants of Health.

^+^Nonparametric Kruskal-Wallis test used

^a^The WellRx total score was used to determine SDOH risk level. One question was added to the WellRx asking about access to physical and mental health care to meet the Social Determinants of Health domain of health care access and quality. One question on access to education was omitted as all students are currently enrolled in a graduate or doctoral program. Low = WellRx scores of 0; Moderate = WellRx scores of 1–2; High = WellRx scores = 3+

*No money can’t buy happiness, money buys resources which I think like would keep my belly happy and shelter to keep me warm, so I don’t freeze. (#9, High, SFI:63)*

*It definitely takes away the experience. I think if I did not have to work, I would have more time to maybe go to the social or go to that conference that they were offering. I would put more of that time into the whole experience of college. (#7, High, SFI:83)*

However, the largest difference was seen in the domain of Financial and Material Stability, where students in the low-risk group had mean domain scores almost twice as high as those in the high-risk group (7.1(SD ± 2.6) versus 3.9(SD ± 2.3)) indicating significantly less domain flourishing with increased social needs (p < .001). Students in the low-risk group also applied a smaller percentage weight to this domain than the high-risk group (14.3%(SD ± 8.2) versus 19.0%(SD ± 10.2); p < .01), indicating a larger perceived impact of financial and economic security on their overall ability to flourish with increased needs. One student in the low-risk group who weighted Financial and Material Stability at 10.0% shared:

*I have a lot of financial support from my husband, so that doesn’t, I guess, affect me as much. (#8, Low, SFI:83)*

#### Health care access and quality.

Over 60.0% of students (22/34, 64.7%) across all three SDOH risk groups shared the benefits of seeing a mental health professional. Fifteen of these 22 students (68.2%) chose, at least initially, to use university-based providers or student support programs, citing convenience, affordability, and the benefit of speaking with a provider who has a strong understanding of the program demands:

*I started therapy, I guess, a little over a year ago now, and the school pays for one therapy session a week, no questions asked, for any graduate student, which is really, really generous. (#16, Low, SFI:103)*

However, 13 of these 22 participants (59.1%) expressed frustration with university services or sought care outside the university system as they had already established care, had more flexibility in appointment options, or had a negative experience with prior attempts to access university care, including a lack of connection with the provider. Additionally, students commented on the lack of racial and ethnic diversity among university health care providers as a barrier to seeking mental health care.

*It’s important, if you need a counselor, therapist, that that person has to match and be able to give you what you need in that situation. And I think me being a Black woman, finding somebody who can offer me the things that I need was hard. (#7, High, SFI:83)*

Seven (7/34; 20.6%) participants also shared concerns regarding stigma of seeking mental health care:

*But I think that becomes more of a concern even with mental health, because that is more sensitive [than physical health]. And I think students are more sensitive to that possibly affecting their academic standings for things, even though there’s policies that say that it doesn’t, but there’s, like, people have biases and things. (#13, Low, SFI:72)*

Despite the positive experiences many students shared regarding their mental health care, over 18.0% of students taking the survey (51/280; 18.2%) responded that they have difficulty accessing the mental or physical health help they need. In the interviews, participants provided additional details about these access-related challenges, which primarily centered around a lack of time, the cost of receiving care, and insurance difficulties. Insurance difficulties included barriers such as no medical insurance provided by the university, inconvenient provider options secondary to Medicaid coverage, and gaps in dental coverage:

*And it’s really like – you know, you’re being blown over by the fire hose like five miles down the road and really, really struggled. And they could not get in for like ten weeks. (#10, Low, SFI:103)*

*I use healthcare.gov, that’s how I have health insurance. It’s not great, but it’s health insurance. I can stay in the program, just don’t get sick, don’t go to the hospital. Those are the rules. (#21, High, SFI:78)*

For students who have chronic health conditions or disabilities, there is an added layer of challenge when not only securing and paying for the necessary health care, but navigating a learning environment that is often divided between the patient and the provider:

*And like for me, that’s like being in medical spaces where we talk about patients being sick and patients being disabled. And we never talk about providers being sick or providers being disabled. Like I have had attendings and fellows and residents talk about patients who have the same condition as me and be like, “wow, it must be really hard to deal with that.” And it completely like what you call disconnected way where like and I don’t really want to out myself and be like, “yeah, that’s what I deal with literally every day.” (#26, High, SFI:53)*

#### Economic stability.

Over one-third of surveyed students (104/280, 37.1%) reported having to work during training, while 33.9% (39/280) reported having difficulty paying for utilities and 9.6% (27/280) reported food insecurity in the past two weeks. In the interviews, students discussed many initiatives to provide financial assistance, including those to address food insecurity on campus:

*I do know that a lot of us in the program benefit from that free meal program and stuff like that, so the program, or, the students in my program have been really good at finding and sharing, disseminating, like, food pilot programs and stuff like that. (#1, Low, SFI:47)*

Of the 104 students who reported having to work during training, the majority (69/104; 66.3%) shared that they are unable to work as much as they need to and 49.0% (50/104) believe work negatively impacts their education. Interviewees reinforced this same sentiment, expressing a lack of financial fulfillment and a daily balance between trying to meet both their financial and academic needs:

*And then on busy days or midterm weeks, like, that was difficult having to, like, be at work and all I wanted to do was study. (#17, High, SFI:88)*

Participants also discussed how these financial concerns are often an ever-present stressor in the background, requiring constant attention and adjustments throughout the day, negatively impacting other domains of flourishing:

*But there’s a sacrifice and I think you have to expect that if you’re going to do something like this, it’s not always fun and you don’t always have time for your family. So saying yes to something and saying no to something else. So if I’m working it’s time I don’t get to spend [with] my boyfriend or my dog or my family or cleaning my own house. (#21, High, SFI:78)*

Students frequently shared their faith that their efforts and sacrifices now will lead to financial stability in the future, but it was not without the realization that the path to this end goal is often ripe with challenges:

*Even though you know you’re working towards being a doctor and you know you’ll eventually get a paycheck or things are going to be okay in the future, you’re living in the now, which is really, really hard because you can’t exactly eat the future for dinner if you don’t really know what it’s going to be today. (#22, Moderate, SFI:71)*

Regardless of unmet economic needs, half of students (17/34, 50.0%) interviewed expressed immense gratitude for their position and the opportunity to pursue their education, frequently using terms such as “lucky,” “blessed,” and “privileged”:

*So – and I still was in a better position than a lot of people. I can recognize my privilege in that, really. (#14, Moderate, SFI:106)*

#### Neighborhood and built environment.

In the survey, 7.5% (21/280) of students admitted to having difficulty paying for necessary transportation. Interview participants shared that transportation needs were largely influenced by the surrounding neighborhood, access to public transportation or university-provided shuttles, and clinical site placements. While no students indicated concerns for homelessness on the survey, rising rent costs and being placed at clinical sites distant from home or campus were a significant source of emotional and financial stress. Students shared there was often a tradeoff between time and affordability of transit options, as taking university shuttles, while viewed as inconvenient and slow compared to having your own vehicle, were often at no cost.

*The difficult part is that you have to invest in the time to get there. So, maybe if I want to go to the VA I’ll take the shuttle from my apartment to the med school, which probably is 20 minutes to 30 minutes depending on when the shuttle arrives and how you get there. And I’ll hop on another shuttle that’s another 30 minutes per se then to go from the medical school to the VA. (#22, Moderate, SFI:71)*

This reliance on needing a vehicle and the lack of convenient public transportation options negatively impacted student flourishing:

*I think when you’re worried about, “Okay, car. Just make it today” – you know? That puts extra stress on you and that’s something else you’re worried about and not so much focus on school. (#10, Low, SFI:103)*

In addition to transportation concerns in the community, and the financial aspect of food insecurity discussed in the subtheme of Economic Stability, students also shared concerns about the inaccessibility of healthy food options surrounding their universities:

*It’s kind of a food desert here, where buying groceries is way more expensive here than it is in a lot of other places, especially if you don’t have a car. (#16, Low, SFI:103)*

Students shared that student loan limits were often not appropriate for the rising cost of living in their community and that they were also not increased to accommodate the added financial burdens that came with clinical rotations, such as paying for duplicate housing at distant sites:

*But you’re sending them to, like, Virginia where they’ve got no family, no support. They’ve got to drive there and pay for a place at $3,000 for, like, an Airbnb for a month...I mean, that’s half their student loan money gone within just like two months. And they still have like five months ago. (#10, Low, SFI:103)*

*Yeah, I mean I don’t really know what the solution would be, but I think asking unemployed full-time students to pay for two rents is like a little bit outrageous. (#6, Moderate, SFI:61)*

#### Social and community context.

Interpersonal relationships provided support for students across all SDOH risk groups. Participants shared the often unwavering support received by peers in their program, the larger medical or nursing communities, and their family and friends throughout training. The camaraderie between peers was shared to be strengthened by expressing vulnerability and the recognition that they are not alone on this journey:

*I think it’s having people to confide in and to learn from, you know, to talk about what you’re going through on an informal basis. And then, like, it’s so helpful to hear what other people have seen, what they’ve gone through too. So, you know, you’re not alone. (#20, High, SFI:76)*

Relationships with individuals outside of the medical or nursing community were also a critical source of support for students. For many, this was through family, long-time friendships, or participation in religious organizations. While students noted the change in time they had to spend engaging in these interactions, many did not believe this impacted the strength of their relationships:

*So, yeah, it absolutely does impact it, but I think that it’s something that I sort of knew and that I’m okay with, I guess, to some extent. Because I do think that there’s, like, even though the time is less, it hasn’t necessarily impacted the strength or the quality, overall, of those relationships. (#25, Low, SFI:104)*

For others, the time commitment necessary for training, paired for some with geographical distance from friends and family during school, provided additional stress on these relationships:

*Yeah, I think it’s just like a completely new environment, so you kind of have to start from scratch, essentially. You’re away from your support systems in a different way and you’re occupying your day in a completely different way, you’re living with new people, you’re, you know, your routines are all thrown off. So it’s just a matter of, like, I don’t think much of it was able to be preserved, and you almost had to kind of start from scratch. (#1, Low, SFI:47)*

In addition to the time and distance barriers, students also shared that it can be difficult for friends and family to relate to or understand the challenges that come along with training, which has negatively impacted their relationships:

*He [husband] doesn’t – he’s not in professional school, and he only heard about how difficult it is. But also me not being able to spend time and care for my child as much as he did make him not understanding why school can be so demanding. Because he’s just not in it to see it. (#12, High, SFI:53)*

In the survey, 14.9% (41/275) of students admitted to seriously considering leaving training in the past six months (Table 1):

*And I think most students, if they were honest, would say at least once a week they’re like, “Why am I doing this? Like, this is what I want to do. Why am I putting myself through this?” Because it does weigh on you. (#10, Low, SFI:103)*

Of these 41 students, 26.8% (11/41) indicated that a lack of connection to the program contributed to this consideration. These same feelings were echoed in the interviews, where students shared feelings of loneliness and isolation throughout training, with more disconnect felt during the clinical phases:

*So, you know, when you’re going throughout the clinical year in some ways, it feels like you’re going through it alone, which is tough. (#33, High, SFI:61)*

#### Education access and quality.

In the interviews, students shared many challenges with accessing medical and nursing education, including the high tuition costs, opportunity cost of not being compensated for the long clinical hours, and accruing student loan debt:

*I think while we’re enrolled the biggest cost, like the biggest reason why we can’t access these things is the amount that we’re paying in tuition and there’s also the opportunity of cost of like not working and so much of medicine is working, but we’re all paying tuition. Like we are paying to work and we are gaining by learning, but it is time that you can’t be doing something else. (#20, High, SFI:76)*

Three NP students (3/9, 33.3%) shared that scholarships were often not available for part-time students, but because of the need to work for financial stability, they were unable to attend full-time. Other students explained that necessary employment made them ineligible for funding that were needs based or that working prevented them from volunteering, another common scholarship requirement:

*And then also, a lotta scholarships require a community service aspect. I love community service. I once did community service all the time. But now I have to take care of my family, I have to go to work, I have to do school. I don’t have time for community service. So now that section of that application is now blank for me, and most likely won’t get it. (#7, High, SFI:83)*

Participants shared that relocating to attend a program brought additional social and financial challenges. For others, the ability to remain in their home community and complete classes online is what made it possible to attend:

*I probably wouldn’t have even been able to apply, because having to sign that contract at the beginning that said “You will have to travel somewhere,” I would have been like “I can’t. Never mind. I guess I can’t accept my seat.” So, it would have changed whether I even applied to PA school probably….This would have been a dream that always just sat on the shelf forever. (#14, Moderate, SFI:106)*

Both online and campus-based students shared their appreciation for learning environments that fostered collaboration and limited competition among peers. Program policies such as pass/fail grading structures also contributed to environments that promoted a sense of balance between personal interests and academic growth, allowing more autonomy of choice in what and how to study. While faculty were frequently cited as co-contributors to the development of learning environments, these contributions were not always positive:

*We get a lot of pressure, a little light on the encouragement side. (#26, High, SFI:53)*

It was often the small actions by faculty, the modeling of vulnerability and empathy, or the extra check-ins, that made the greatest impact:

*And they all start – the first module, the welcome – at the beginning of the semester, they all have started by saying, “Please communicate with us. Let us know. We understand life happens. You’re working. People have kids and husbands and life occurs. And so – and we understand because we too have experienced at one point trying to balance the demands of life.” (#2, High, SFI:46)*

Additional representative quotes from each SDOH subtheme can be found in table 4. In the end, it was an overwhelming commitment to their future professions that carried students through the hardship across all three SDOH risk levels, with many relying heavily on their intrinsic motivation for pursing their respective careers and their foundational love of learning:

**Table 4:** Representative quotes for social determinants of health subthemes.

Health Care Access and Quality	*But I think it really helps to have someone who is attached to the school who knows what we're going through because when you go in and you try to explain that you just cut up someone's pelvis today because you're learning the bones you wouldn't have to also justify the things that you're doing because they're aware of your different curriculums. (#22, Moderate* ^*a*^ *, SFI: 71)* *This is, to be clear, I'm an MD-PhD student and so we get [University] health insurance provided for us. I'm not sure how it works if you don't have student health insurance, because like we get the full student health insurance that you have to like buy into, provided as part of our like stipend package. So I've never had to think about health insurance here, which honestly has been really nice for me. Part of why I decided to come here. (#26, High* ^*a*^ *, SFI: 53)* *For example, I have a tooth that got broken and I called almost 20 clinics; none of them accept Medicaid. (#11, Low* ^*a*^ *, SFI: 63)* *It becomes harder to access when you're clinically active, like, because you're working the entire time when you have appointments available. And so, it's just hard to step out and have that time just to be able to access those appointments. (#13, Low* ^*a*^ *, SFI: 72)*
**Economic Stability**	*I try to maintain this employment, but at the risk of, like, again, now I'm feeling like I'm burning out, I don't know how I was really managing all this, even back then, maybe because it was more flexible, but the clinical year, it's just, you know, you already work a full day, and then going home and doing more work is just exhausting. (#33, High* ^*a*^ *, SFI: 61)* *I'm always just thinking about like, “Oh, I probably shouldn't buy the more expensive bread,” you know, like you're always like making these like small, little changes just to try to conserve your money as best you can throughout the semester. (#6, Moderate* ^*a*^ *, SFI: 61)* *“Maybe I need to pick up an extra shift” or – and so, then it's “Well, but I really need this time to study. But I need the money”. (#2, High* ^*a*^ *, SFI: 46)*
**Neighborhood and Built Environment**	*I grew up in a city. I wanted to live in a big city. I love public transit because being disabled, my disability makes it hard for me to drive. Um, and so like it was a sacrifice for me and favor of health insurance to come here rather than a program in a bigger city. (#26, High* ^*a*^ *, SFI: 53)* *I know that just a few students have gone back to like financial aid and asked for, you know, and increase and they got maybe like $500 like, “Well, that $500 still isn't going to, you know, pay my rent – you know, cover the rest of my rent.” (#10, Low* ^*a*^ *, SFI: 103)* *I don't know if this is for all programs, but we can apply for like AHEC housing, so I think that's reduced cost living or maybe free, I'm not sure. But my issue there is I have cats so I can't just leave them here for a month. (#6, Moderate* ^*a*^ *, SFI: 61)* *Yeah, that's actually a problem within it as well. So I have a psych rotation in Orangeburg coming up and that's like an hour-and-20-minute drive. I would ideally like – I would have liked to be able to take my wife and child, but they only provide a room and they don't provide any guests. I don't think they allow pets either. (#19, Moderate* ^*a*^ *, SFI: 105)*
**Social and Community Context**	*A example – last semester, I was – it was almost done. And I had people in my group that, without me saying, was like, “Oh, something must be going on with [name],” and reached out to – sent a e-mail, made a call. I'm like, “Wow.” I wouldn't have expected that. (#7, High* ^*a*^ *, SFI: 83)* *And I have my core group of friends that they're not PAs or they don't have grad school degrees, but they just understand that I'm not always available. We have that kind of friendship where it's very low maintenance. (#21, High* ^*a*^ *, SFI: 78)* *It's definitely really difficult. There are aspects that are especially like caring for other people like sometimes I am just surviving and I don't have a lot of bandwidth for other people. Sometimes it's hard for me to feel like I'm even loving my wife and my baby well. (#19, Moderate* ^*a*^ *, SFI: 105)* *So it's hard to find time to see anyone outside of my med school friends. And then too, it's hard for them to relate to what I'm doing, and exactly like what the demands are of it. (#32, Low* ^*a*^ *, SFI: 97)* *Yeah, for sure. I think isolation is… I mean since community is a big part of flourishing, isolation is the big part of not doing well. I've seen some students in my cohort dropout because they're struggling, but they're isolating because they're afraid to share the ways that they're struggling with people and so they just get to a point where they can't keep going. (#19, Moderate* ^*a*^ *, SFI: 105)*
**Education Access and Quality**	*It's all just such a freaking racket. They're just – it's like “Hey, we want people to have higher education and to help America be amazing, but at the same time we're going to make you so far in debt that you'll never crawl out from under it.” So, yeah, it's difficult. (#14, Moderate* ^*a*^ *, SFI: 106)* *Because you're – I'm only one person. There's only so much that I can do. And if there is not resources that I can tap in to assist with those, then it can't be done and still be a functional person. So if my needs were higher, I don't think that this would be an option for me. (#7, High* ^*a*^ *, SFI: 83)* *Like if you want to study, you study like what is going on with your patients right now, instead of like, doing all these questions for, like, hours to study for that exam. Yeah, So I think that has had a huge impact on, like, all med students’ lives at [University]. (#20, High* ^*a*^ *, SFI:76)* *If we're in healthcare we do it because I mean we have the grades, we have the, we have the ability, you know, there's a huge vetting process before we get here and now we need support, because we are own worse critics. And sometimes it feels like we get extra criticism from others which can be really unhelpful. (#19, Moderate* ^*a*^ *, SFI: 105)* *Like I said, the competition. I think some of them can't get past the guilt. Like, “No, I should be studying all the time.” I also think there is probably certain, like, faculty members that sometimes probably make you feel like you should be studying all the time. (#10, Low* ^*a*^ *, SFI: 103)* *I mean [name] was my advisor so I felt – Like, I felt – like I remember he sent me a card on my birthday and I was like, “Wow.” It was just, like, so above….Like, that made me feel like, “Oh. He's really there, not just as a figure being an academic advisor.” (#17, High* ^*a*^ *, SFI: 88)* *So, keeping everything pass/fail lets people put in the focus that they know they need and should, while also giving them the space to spend time elsewhere, if that's going to be more valuable for their growth. (#16, Low* ^*a*^ *, SFI: 103)*

Abbreviations: SFI: Secure Flourish Index

^a^The WellRx total score was used to determine social determinant of health risk level. One question was added to the WellRx asking about access to physical and mental health care to meet the Social Determinants of Health domain of health care access and quality. One question on access to education was omitted as all students are currently enrolled in a graduate or doctoral program. Low = WellRx scores of 0; Moderate = WellRx scores of 1–2; High = WellRx scores = 3+

*If it’s, like, if this is just something that you do to get a paycheck, it just, it takes too much emotionally, mentally, you know, and probably even physically, to some extent, to just do that. (#25, Low, SFI:104)*

## Discussion

This explanatory mixed-methods study found that medical, NP, and PA student flourishing varies by SDOH risk level and definitions of flourishing are highly individualized. However, as a collective, students provided a definition of flourishing that includes personal growth in the presence of both happiness and success. When paired with living experiences and unmet social and economic needs, the nuanced variations in definitions shaped the individual perceptions of the relative importance of established flourishing domains. This relationship between individual values and perceptions of flourishing is missing from current operant definitions [12].

Previous analysis has shown that applying a relative weight to the domains of flourishing using the SFI produces a flourishing score that is significantly different from the traditional scoring approach [30–31]. Our mixed-methods analysis allowed for the exploration of factors considered when applying these weights to the flourishing domains, which was previously unknown. We found that while two students may have the same fulfillment or security in a domain, this may cause one to weight this domain highly important to their ability to flourish while another may consider it minimally impactful. For example, two students may report similar flourishing in the domain of Close Social Relationships, but only one finds these relationships foundational to their ability to flourish. This difference in perception highlights the need for faculty to account for these differences in advising discussions and wellness interventions. Additionally, the use of SDOH provided a framework for exploring a more holistic understanding of student perceptions of not only flourishing, but also basic needs.

Our findings are consistent with prior literature which has shown that unmet social and economic needs are prevalent throughout the medical and nursing student communities [32–35]. The interviews conducted in this study provided context to these prior findings, exploring the role of social relationships and support systems, university structure, the surrounding community, and public policy in student flourishing. While economic security is its own determinant, the financial stressors faced by many students throughout training intermixed with the other determinants. Students expressed needing to work for living expenses but also to maintain health insurance, a university requirement but not something provided by every program. This need to work was found to negatively impact participation in academic-related extracurricular activities and impede the ability to engage in social events, spend time with family, or exercise, all components of the SFI domains and SDOH. While programs often encourage students not to work during training, this is not a realistic option for many students. Additionally, factors such as insufficient student loans access, high interest rates, and rising rent costs were further exacerbated when programs were unable to secure local clinical rotations sites, requiring students to commute long distances without access to reliable transportation or to secure housing in multiple locations at once.

This intermingling of the SDOH and SFI domains caused many students to feel financial stress that had a broad negative effect on their ability to flourish in multiple areas of life; however, others accepted this strain as a part of training and therefore reported feeling very little effect on their current sense of flourishing. This varied perspective reinforces the need for a more individualized approach to wellness and intervention [36]. Additionally, screening for either SDOH or flourishing without considering a weighted approach as employed in this study may fail to identify the myriad of challenges and interactions that negatively affect students’ ability to flourish. Integrating discussions of flourishing domains and SDOH into advising sessions may help guide suggestions for support; for example, recognizing that a student feels they are lacking in the domain Close Social Relationships may prompt facilitation of a formal mentoring relationship or connection with community service groups. Our findings provide support for creating more personalized wellness interventions that are created to target high priority domains for individual students.

In 2022, Surgeon General Dr. Vivek Murthy stated that the health of Americans is dependent on the well-being of the United States healthcare workforce [37]. With nearly half of all health care employees facing burnout and considering leaving their jobs in the next decade, reducing burnout and promoting flourishing is a critical piece of the nation’s healthcare infrastructure [37]. This study provides insight for the development of an operant definition of flourishing that matches each individual’s values and simultaneously highlights areas for personalized interventions, a current gap in existing instruments. Faculty should consider SODH in addition to factors outside the classroom at each level of the SEM when developing wellness interventions. Practically, this may include facilitating flourishing-centered advising discussions, establishing emergency funds for unexpected student expenses, securing affordable housing or scholarship funds for distant clinical rotation sites, and integrating flexibility into student schedules to accommodate use of university mental and physical health services.

### Limitations

The quantitative sample size was below the target; however, data saturation was achieved with interviews. Given the nature of the discussions surrounding mental health and social or economic needs, participants may have self-selected out of participation or may have adapted the information shared in interviews. Additionally, the relationship between race, SDOH, and flourishing was not explored; this warrants future investigation given the intersection of the social and structural determinants of health. As researchers were affiliated with both institutions, students may have been dissuaded from participating.

## Conclusion

This study found that SDOH risk significantly impacts student flourishing, with high-risk students reporting SFI scores over 15 points fewer than low-risk students. Additionally, high-risk students assigned nearly 5% more weight to the domain of Financial and Material Stability than low risk students, amplifying the perceived importance of this domain as SDOH risk increases. While individual definitions of flourishing vary, the majority of students view flourishing as a balance of happiness and success. Perceptions of flourishing and the relative importance of established flourishing domains are highly influenced by the social determinants of health and living experiences. Educators should consider these individual perspectives and values when measuring flourishing and planning wellness interventions.

## Supporting information

S1 File Complete survey questions. This file contains the complete survey used in this study.
(PDF)

S2 File Complete interview protocol. This file contains the complete interview protocol used in this study.
(PDF)
