# Supplementary material for: An exploration of perceptions of flourishing and social determinants of health among medical, physician assistant, and nurse practitioner students: A mixed methods study
Source: PLoS One. 2026 Feb 25;21(2):e0343630. doi: 10.1371/journal.pone.0343630 (PMC12935265; doi:10.1371/journal.pone.0343630)
Supplement: S2 File — This file contains the complete interview protocol used in this study. (PDF) [file pone.0343630.s002.pdf]

Interview Qualitative Protocol

Semi-Structured Interview Prompts:

Throughout these questions, I will refer to your "training." For the purposes of this interview, training should include your didactic coursework as well as your clinical experiences. This should go back to when you first enrolled in your current program, but not extend to degrees or clinical training prior to beginning your current program. For example, if you were an EMT prior to beginning your current PA program, your "training" here refers to your time in your current PA program, not your time served as an EMT.

Flourishing Perspective and Experience:

- How do you define flourishing? What do you view as the difference between happiness, flourishing, and success?
    - What do you think helps students flourish during training? What makes it harder to flourish?
- What parts of your current training or academic program give you joy? What parts of your personal life give you joy? Why?
    - If nothing, has this changed since beginning training or your program? Why do you think this is the case?
- What do you do to promote your own personal flourishing?
    - Is there anything that helps you?
    - Is there anything that impedes this?
- What does your school (or program) do to intentionally promote flourishing among students, if anything? Describe the types of things that they offer individuals. These might be like mindfulness training or something similar or the efforts of a specific faculty member within a course. What other types of programs or services do they offer within your program or institution that could help students flourish? These might be things like yoga classes or other types of wellness programs.
- What recommendations to you have to improve or enhance these? What, in your opinion, is most needed or currently lacking?

Social Needs:

- In the survey, you weighted [*share domain*] as being the most influential in your ability to flourish; what were some of the things you considered or were thinking about when deciding how to weight this domain and how much to allocate to the other domains of flourishing on the survey?

- For the areas that you rated the most important [share individual results] to your ability to flourish, how well do you feel that your needs in these areas are currently being met? How much of a factor do you think it is that you are currently a student when you think about how you weighted these different domains?
- For the areas that you rated the least important [share individual results] to your ability to flourish, how well do you feel that your needs in these areas are currently being met? How much of a factor do you think it is that you are currently a student when you think about how you weighted these different domains?

- When we think about social needs, we are referring to things like having access to housing and health care, along with having food security. How much of a factor are these types of social needs on your ability to flourish, in your opinion? If your social needs [e.g., housing, food security, access to health care] were different, higher or lower, how do you think this could affect your sense of flourishing?
  - How, if at all, have policies regarding financial aid eligibility limits, interest rates, and repayment programs affected your ability to flourish during training?
  - What kinds of social needs support, if any, is available for students at your program/university?
  - Many students indicated current needs such as for healthcare, housing, financial support, and other areas to help them survive day to day. What do you think, if at all, is your programs or university's responsibility in helping you meet these needs while enrolled as a student?

- *For students who indicated >0 social needs:* How has your need for [social need(s)] impacted your ability to flourish through training? How do you believe this has influenced your academic performance? How has this impacted your ability to participate in optional or extra-curricular activities? How has this impacted your relationships and connections with your peers? What ideas do you have that may help future students in your same situation?

Grit and Coping:

- How do you cope with the challenges of training? Challenges in your personal life?
- When training becomes difficult, what motivates you to continue? Please specify didactic versus clinical phases of training.
  - How do you cope with challenges in your personal life? To what extent has this changed since you began your program?

- When thinking about your academic program or training, what has either increased or decreased your desire to stay in the program?
  - Why do you think some students consider leaving training? What recommendations do you have to help students remain in their training programs?

- What role do you think friendships and a sense of community or belongingness among your peers play in flourishing during training?
    - What does your current support system look like? How has it changed at different stages of training? What types of things affect your ability to connect with others?
    - What do your friendships within the program look like? What about outside of your program? How has training impacted your relationships? Tell me about what your support system looked like before training?
- What role do you think spirituality or religion play in flourishing during training?

Closing:

- If you could change one thing about your training experience to improve your own flourishing, what would you change?
- What else is important that I may not have asked about that we should consider in looking at student flourishing?

Additional questions considered:

- What experiences have you had as a student that have either increased or decreased your sense of flourishing?

- Please share any details about your current school-life balance?

- Tell me about a time when you believe you had strong work/school-life balance? Why do you think this balance occurred? What has changed and why do you think this occurred?

- What types of program or university wellness efforts have you participated in? What were your thoughts about this/those? What do you think could be improved regarding wellness programs offered?

THANK YOU FOR TAKING THE TIME TO HELP US WITH THIS PROJECT!
